# High genomic diversity in the endangered East Greenland Svalbard Barents Sea stock of bowhead whales (*Balaena mysticetus*)

**DOI:** 10.1038/s41598-022-09868-5

**Published:** 2022-04-12

**Authors:** José Cerca, Michael V. Westbury, Mads Peter Heide-Jørgensen, Kit M. Kovacs, Eline D. Lorenzen, Christian Lydersen, Olga V. Shpak, Øystein Wiig, Lutz Bachmann

**Affiliations:** 1 Natural History Museum, University of Oslo, P.O. Box 1172, 0318 Blindern, Oslo, Norway; 2 GLOBE Institute, University of Copenhagen, Øster Voldgade 5-7, Copenhagen K, Denmark; 3 Greenland Institute of Natural Resources, Strandgade 91, 1401 Copenhagen K, Denmark; 4 Norwegian Polar Institute, Fram Centre, 9296 Tromsö, Norway; 5 A.N. Severtsov Institute of Ecology and Evolution of Russian Academy of Sciences, 33 Leninsky Prospect, Moscow, Russian Federation 119071; 6 Present Address: NTNU University Museum, Norwegian University of Science and Technology, Trondheim, Norway; 7 Present Address: Independent scientist, Kharkiv, Ukraine

**Keywords:** Genomics, Population genetics, Molecular evolution, Ecological genetics, Ecology, Evolution, Genetics

## Abstract

The East Greenland-Svalbard-Barents Sea (EGSB) bowhead whale stock (*Balaena mysticetus*) was hunted to near extinction and remains *Endangered* on the International Union of Conservation of Nature Red List. The intense, temporally extensive hunting pressure may have left the population vulnerable to other perturbations, such as environmental change. However, the lack of genomic baseline data renders it difficult to evaluate the impacts of various potential stressors on this stock. Twelve EGSB bowhead whales sampled in 2017/2018 were re-sequenced and mapped to a previously published draft genome. All individuals were unrelated and void of significant signs of inbreeding, with similar observed and expected homo- and heterozygosity levels. Despite the small population size, mean autosome-wide heterozygosity was 0.00102, which is higher than that of most mammals for which comparable estimates are calculated using the same parameters, and three times higher than a conspecific individual from the Eastern-Canada-West-Greenland bowhead whale stock. Demographic history analyses indicated a continual decrease of *N*_e_ from ca. 1.5 million to ca. 250,000 years ago, followed by a slight increase until ca. 100,000 years ago, followed by a rapid decrease in *N*_e_ between 50,000 and 10,000 years ago. These estimates are lower than previously suggested based on mitochondrial DNA, but suggested demographic patterns over time are similar.

## Introduction

The bowhead whale (*Balaena mysticetus*) is the only baleen whale that lives its entire life in Arctic and subarctic regions, often in association with sea ice^1^. Four bowhead whale stocks are currently recognized: (1) the Bering/Chukchi/Beaufort Seas (BCB) stock; (2) the Okhotsk Sea (OKH) stock; (3) the Eastern Canada-West Greenland (ECWG) stock; and (4) the East Greenland-Svalbard-Barents Sea (EGSB) stock, earlier referred to as the Spitsbergen stock^2^. The bowhead whale, as a species, is listed as *Least Concern* in the International Union of Conservation of Nature (IUCN) Red List. However, the EGSB stock, which is distributed from the East Greenland Sea across the northern Barents Region into Russia (including Severnaya Zemlya and Franz Josef Land waters), is classified as *Endangered*^2^.

There has been ongoing discussion regarding the census size of the EGSB stock. This population was thought to be large prior to the onset of extensive hunting, which commenced circa 1611. Estimates of the preharvest stock size range from 25,000 to 100,000 individuals^3^. When hunting ceased in 1932, the population was thought to be depleted to near extinction^4,5^. Recent estimates of the current size of this stock have ranged from a few tens^6^ to several hundred individuals^7^. In support of these low estimates, only a few bowhead whale sightings were reported from ship-based surveys in the Fram Strait during spring 2006, 2008 and 2010^8–10^. A combined helicopter- and ship-based line transect covering the marginal ice zone (MIZ) from the Russian border westward to the northwest corner of the Svalbard Archipelago estimated some 350 individuals in this region in late summer^11^. Surveys conducted in East Greenland waters in spring and late summer/early fall documented similar numbers in the western parts of the stock’s range^12^. However, individual animals move across the whole range; bowhead whales tagged in East Greenland shelf waters have travelled eastward to Franz Josef Land and beyond^1^.

Several genetic studies have focussed on elucidating the genetic structure and differentiation of the four recognized bowhead whale stocks^13–17^. For the EGSB stock specifically, four studies, all targeting mitochondrial markers, have been conducted. Borge et al*.*^14^ assessed mitochondrial haplotype diversity by sequencing parts of the mitochondrial D-loop region in bone remains from 99 historic bowhead whales. They found relatively high genetic diversity with levels similar to the genetic diversity of the extant BCB stock, which has also been depleted by extensive hunting. Three further studies generated full mitochondrial genome sequences from historic^18^ and contemporary individuals^17,19^. The contemporary mitochondrial genome sequences supported the earlier findings of Borge et al*.*^14^ that the EGBS population is genetically differentiated from the other three stocks. Inferences regarding the demographic history of the EGSB bowhead whale stock suggested a boom–bust scenario that included Late Pleistocene population growth 25,000–35,000 years ago, followed by a Holocene decline^17^. However, the confidence limits of the estimates were broad and overlapping throughout the time frame of the study, making it impossible to define numerical trends for this population. Since mitochondrial DNA is a single, maternally inherited, non-recombining marker, and thus particularly sensitive to drift in small populations, inferences based on just mitochondrial DNA have to be interpreted cautiously, as the data may not fully represent the diversity and demography of the EGSB stock. Accordingly, it is important to obtain nuclear genomic data that offer more refined insights.

The current study addresses levels of genetic diversity in the EGSB stock of bowhead whales based on nuclear genomes from the 12 individuals that had been previously analysed using mitochondrial genomes^17^. Nuclear high-throughput sequencing data were analysed for heterozygosity, population structure, inbreeding and demography. The heterozygosity estimates were compared to those obtained for archived sequence data for a conspecific individual from the ECWG stock and a North Atlantic right whale (*Eubalaena glacialis*). These genomes and the findings provide important baseline data for future conservation and management of bowhead whale stocks.

## Materials and methods

### Ethics declaration

Permits for animal handling were issued by the Norwegian Animal Research Authority (FOTS ID: 11,821), the Governor of Svalbard (Sysselmannen, permit ID: 16/01,600–6) and the Greenland Ministry of Fisheries, Hunting and Agriculture (ref. 2017–2551, akt. no.8267820). All methods were carried out in accordance with relevant guidelines and regulations. All methods are reported in accordance with ARRIVE guidelines (https://arriveguidelines.org) for the reporting of animal experiments.

### Sampling

**Figure 1:**
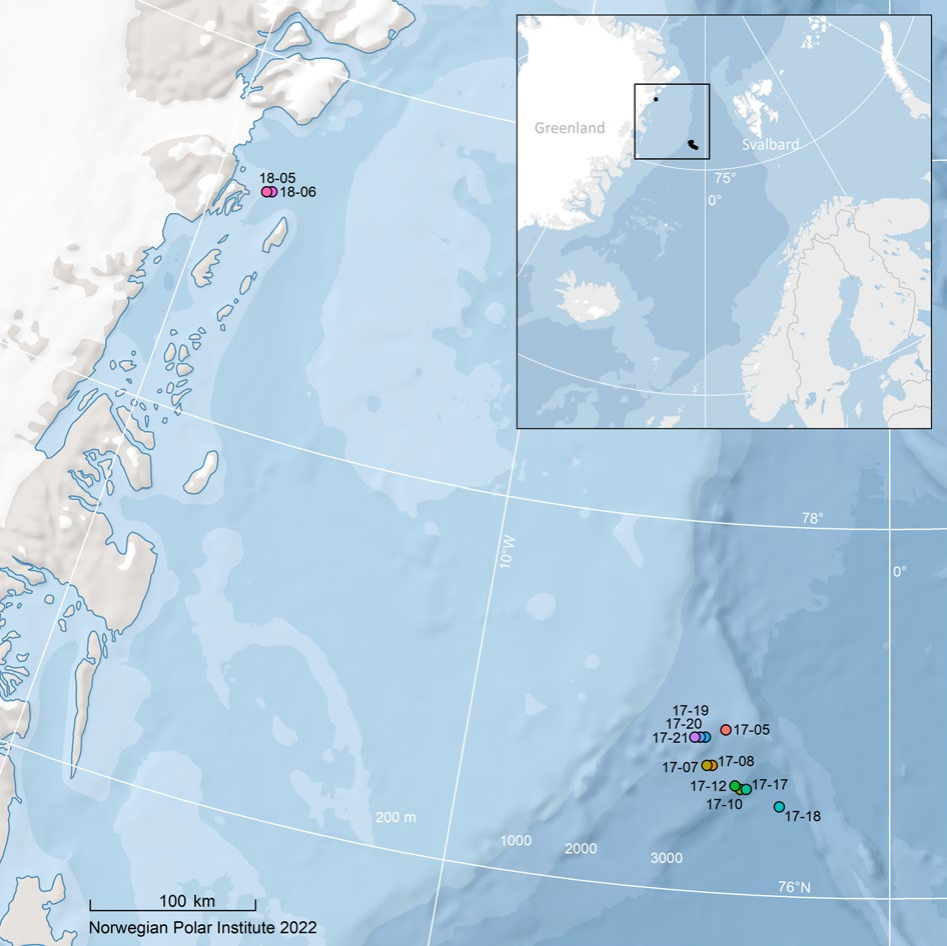
Sampling locations of the bowhead whale skin biopsies used in this study. The map was generated based on publicly available ArcMap polar projections documents using ArcGIS 10.1 (www.esri.com).

Twelve bowhead whale skin biopsies collected during field expeditions in 2017 (ten samples) and 2018 (two samples) using a crossbow and custom-made darts were used in this study (for details see Bachmann et al*.*^17^). The sampling sites are depicted in Fig. 1. In addition, archived genome sequence data were downloaded from seven bowhead whale samples collected in 2006 and 2010 (for details see Nyhus et al*.*^17^, GenBank Bioproject: PRJNA798027).

### DNA extraction and sequencing

Total genomic DNA was extracted using the E.Z.N.A. Tissue DNA kit (Omega Bio-Tek) following the Tissue DNA-Spin Protocol provided with the kit. Building of Illumina sequencing libraries and sequenced using 150 bp paired-end read chemistry on an Illumina NextSeq 500 was outsourced to StarSEQ GmbH, Mainz, Germany. The raw reads have been deposited in NCBI's Sequence Read Archive (GenBank Bioproject: PRJNA643010; accessions listed in Supplementary Table S1).

### Sequencing read mapping, variant calling and data filtering

This study aimed at a sequencing depth of > 10 for the 12 bowhead whale samples in order to allow for reliable estimates of genetic diversity. Raw reads were quality checked using FASTQC v0.11.8^20^ and trimmed and filtered for Illumina adapters and low-quality bases using Trimmomatic v0.39^21^ with a default error rate (-e 0.1), an adaptor overlap of 1 (-O 1) and a quality cut-off of 20 (-q 20). Filtered reads were aligned to the *Balaena mysticetus* draft genome^22^ using the BWA-mem algorithm^23^. An overview of the mapping statistics and depth is provided in Supplementary Table S1. The sequences of the resulting alignments were sorted, marked and indexed using Picard tools v2.10.4 (http://broadinstitute.github.io/picard/). The GATK v3.7 (https://gatk.broadinstitute.org/hc/en-us) RealignerTargetCreator and subsequently IndelRealigner tools were used to remove PCR-duplicates and to account for insertion-deletion (indels) polymorphisms, which can affect downstream variant calling. Variant calling was performed both for variant and invariant sites using the GATK v3.7 HaplotypeCaller tool, resulting in a gVCF (genome Variant Call Format file) for each sample. gVCFs were then merged using the GenotypeGVCFs tool, generating a raw VCF file for downstream analyses. This raw VCF file was then filtered for QualByDepth (QD) < 5.0, FisherStrand (FS) > 10.0, RMSMappingQuality < 40.0, MQRankSum < -2.5, and ReadPosRankSum < -2.5. Finally, to include only single nucleotide polymorphism (SNP) variants, the VariantFiltration tool included in GATK and vcftools v0.1.13^24^ was used.

### Population structure and phylogenetic network

A Principal Component Analysis (PCA) and a phylogenetic network were computed from the above variant calls to address population structure, branching pattern and the distribution of individuals within the population. The VCF file generated by the the GenotypeGVCFs tool was further filtered to remove SNPs with a minimum allele frequency < 0.05 and a mean depth value (across all included individuals; > 10 × and < 100x) using vcftools v0.1.13^24^. For the phylogenetic network analysis, the VCF file was converted into phylip format using vcf2phylip v2.0^25^ and the analysis was performed using Splitstree v4^26^. For the PCA, a linkage disequilibrium (LD) filter was applied with a window size of 50,000 bp and a step size of 10,000 (defining a r^2^ threshold of 0.18) using plink v1.90b5.2^27^. This high-quality, unlinked set of variants was loaded into R v3.6^28^ using the vcfR package^29^, and PCA was performed using adegenet^30^.

### Relatedness and inbreeding

Observed and expected numbers of homozygous sites were determined from the GATK filtered variants using vcftools (–het)^24^. Significance of the differences between the observed and expected homozygous values was tested using a Welch two sample t-test. Additionally, individual inbreeding (F) and relatedness (R) levels between samples were estimated using NGSrelate v2, which uses identity by descent to calculate these metrics^31^. In contrast to vcftools, this method does not rely on directly calling genotypes, but instead gives the likelihood that any of the four nucleotides could be called at a certain position. Therefore, this method can also produce reliable results for low-coverage data (down to ca. 1x). Furthermore, NGSrelate relies on a population-level reference panel to compute relatedness and inbreeding, the more individuals included in the analysis—the more reliable the results are. Thus, for the relatedness analysis, the seven samples collected in 2006 and 2010 (previously sequenced to low coverage (0.8× to 2.45×), for sample details see SRA; Bioproject: PRJNA798027; accessions listed in Supplementary Table S1, and Nyhus et al*.*^19^) were also included. As input for NGSrelate, genotype likelihoods (GL) of the dataset were calculated from the mapped bam files using ANGSD v0.921^32^. The following filtering options were used: minimum mapping and base quality of 30 (-minmapQ and -minQ 30); calculate genotype likelihoods using the GATK algorithm (-GL 2); output binary genotype likelihoods (-doGlf 3); infer major and minor alleles using GL (-doMajorMinor 1); calculate per site frequencies using a fixed major and unknown minor allele (-doMaf 2); only include SNPs with a *p*-value less than 1 × 10^–6^ (-SNP_pval 1e-6), and a minimum minor allele frequency of 0.05 (-minmaf 0.05). The number of sites included in the pairwise comparisons between individuals to calculate relatedness ranged from 2,118,337 to 12,161,246. Further, runs of homozygosity (ROH) as a proxy for inbreeding were determined in each of the 12 newly generated genomes (samples collected in 2017 and 2018) using ROHan^33^. The program was run using only autosomal scaffolds > 1 Mb in length to avoid biases due to the fragmented assembly. However, it should be noted that although the analysis still contained 968,853,266 bp of data, ROH may be downwardly biased from the fragmented assembly. ROHan was run twice, once specifying a ROH if 1 Mb of data contains an average proportion of heterozygous sites less than 1 × 10^–5^ and once specifying the average proportion of heterozygous sites less than 5 × 10^–5^. Bowhead whale X and Y-linked scaffolds were identified by aligning the published bowhead whale genome assembly to the cow X chromosome (GenBank accession number: AC_000187.1) and the human Y chromosome (GenBank accession number: NC_000024.10) using satsuma synteny^34^. The latter were subsequently removed because they can bias heterozygosity estimates in males.

### Genetic diversity

Autosome-wide heterozygosity was calculated for the seven samples with > 20 × average read depth (Supplementary Table S1). All individuals were down-sampled to 20 × read depth using SAMtools^35^ in order to avoid coverage linked biases and to ensure comparability between previous estimates. Genetic diversity estimates were subsequently determined using ANGSD v0.921^32^ with the filtering steps following Westbury et al*.*^36^ and only compared against estimates from other species calculated using the same software and parameters. The filtering parameters were as follows: calculated genotype likelihoods using the SAMtools algorithm (-GL 1); only consider sites with at least 5 × coverage (-setMinDepthInd 5); minimum mapping and quality scores were defined as 25 (-minmapq 25 and -minq 25); only consider reads mapping to a single region uniquely (-uniqueonly 1); remove secondary alignments (-remove_bads 1); calculate allele frequencies (-doSaf 1); only use scaffolds > 100 kb (-rf); perform indel realignments (-baq 1); and do not specify an ancestral allele (-fold 1). The genomic raw reads (GenBank biosample ID: SAMN14122067) of the North Atlantic right whale (*Eubalaena glacialis*) were downloaded and mapped to a North Atlantic right whale reference genome retrieved from https://www.dnazoo.org/assemblies/Eubalaena_glacialis using BWA mem and default parameters. From a total of 773,071,534 reads (386,535,767 read pairs) 596,115,335 resulted in a mean read depth of 38.8x. Autosome-wide heterozygosity for the right whale was calculated using the same approach as described above for the bowhead whales.

### Demographic analysis

Deep time demographic analyses (changes in effective population size (*N*_e_) through time) were run on each individual using a Pairwise Sequentially Markovian Coalescent (PSMC) model^37^. As input for PSMC, indel-realigned diploid consensus sequences were built from the mapped bam files using SAMtools^35^ and BCFtools (http://samtools.github.io/bcftools/bcftools.html). The atomic time interval setting 4 + 25*2 + 4 + 6 was used^38^. Mutation rate was set to 2.69 × 10^–8^ substitutions per generation (i.e. 7.69 × 10^–10^ per site per year), a value specifically calculated for bowhead whales by Westbury et al*.*^38^. Following Rooney et al*.*^13^ the generation time was set to 35 years, an estimate that is based on information about lifespan, sexual maturity and reproduction intervals of female bowhead whales. However, generation time of bowhead whales is a topic of debate, and estimates may be considered 'guesstimates'. Other studies^39,40^ have used ca 50 years, and we repeated the analyses with a generation time of 50 years. Different generation times will affect the timelines of demographic analyses but not the overall trajectories. To estimate the more recent demographic history of the EGSB stock stairway plots v2^41^ was implemented using the unfolded site frequency spectrum (SFS) calculated using the 12 newly generated genomes (samples collected in 2017 and 2018). The right whale was mapped to the bowhead whale reference genome using BWA mem to determine the ancestral state. A total of 542,282,179 reads mapped, resulting in a mean read depth of 38.8x. The SFS was calculated from allele frequencies in ANGSD v0.921^32^ (-doSaf 1) from all scaffolds > 100 kb in length with the following parameters: -minmapQ 25, -minQ 25; -GL 2, -doMajorMinor 1; -doMaf 2, only use sites where all individuals have coverage (-minind 12); skip triallelic sites (-skipTriallelic 1); and -setMinDepthInd 5. The same generation times and mutation rate were used as for the PSMC analyses. An analysis using a folded SFS was also run to investigate whether the ancestral state could impact the results. ANGSD was run in the same fashion as for the unfolded SFS but with the -fold option and the -anc sequence replaced with the bowhead whale reference genome instead of the right whale.

## Results

### Variant calling and SNPs

Direct variant calling of the mapped reads across all individuals yielded a total of 11,928,111 variable sites after initial GATK filtering. After further filtering for coverage and minimum allele frequency, and after removing indels, the dataset retained 7,750,241 SNPs. The specific numbers of SNPs for each individual included in the downstream analyses are listed in Table 1. After further filtering for linkage disequilibrium (LD) the SNP set used for the PCA numbered 332,255 SNPs across the 12 EGSB stock samples.

**Table 1:** Observed and expected numbers of homo- and heterozygous sites and autosome-wide heterozygosity proportions for the EGSB stock samples with an average coverage > 20 × calculated with ANGSD^32^.

Bowhead whale	Number of assessed SNPs	Observed homozygous SNPs	Expected homozygous SNPs	Observed heterozygous SNPs	Expected heterozygous SNPs	Heterozygosity proportions
17–05	7,707,648	5,191,417	5,114,415	2,516,231	2,593,233	
17–07	7,713,101	5,100,155	5,117,900	2,612,946	2,595,201	0.00102
17–08	7,717,794	5,063,352	5,120,835	2,653,842	2,596,959	0.00102
17–10	7,711,971	5,209,113	5,117,141	2,502,858	2,594,830	
17–12	7,717,953	5,077,716	5,120,931	2,640,237	2,597,022	0.00102
17–17	7,717,011	5,200,933	5,120,305	2,516,078	2,596,706	0.00098
17–18	7,717,338	5,043,461	5,120,541	2,673,877	2,596,797	0.00103
17–19	7,713,315	5,127,866	5,118,025	2,585,449	2,595,290	0.00105
17–20	7,700,325	5,322,498	5,109,892	2,377,827	2,590,433	
17–21	7,718,136	5,072,339	5,121,074	2,645,797	2,597,062	0.00103
18–05	7,713,449	5,144,031	5,117,923	2,569,418	2,595,526	
18–06	7,714,979	5,105,321	5,118,945	2,609,658	2,596,034	

### Population structure

**Figure 2:**
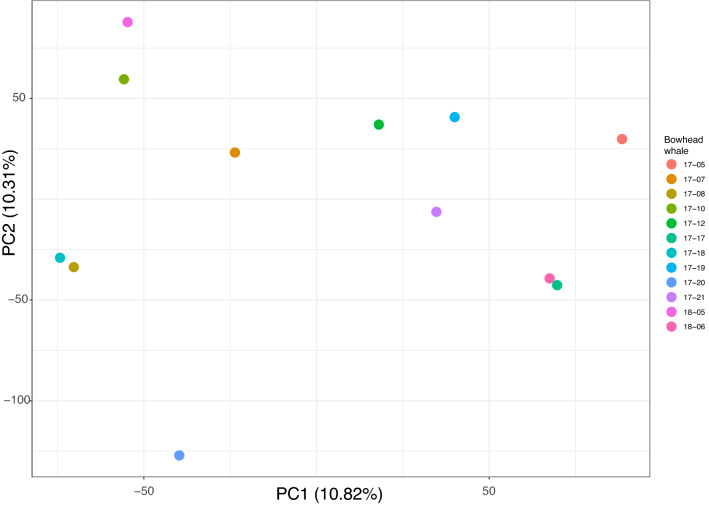
PCA exploring population structure in EGSB stock bowhead whales sampled in 2017 and 2018. No obvious population structuring was identified.

**Figure 3:**
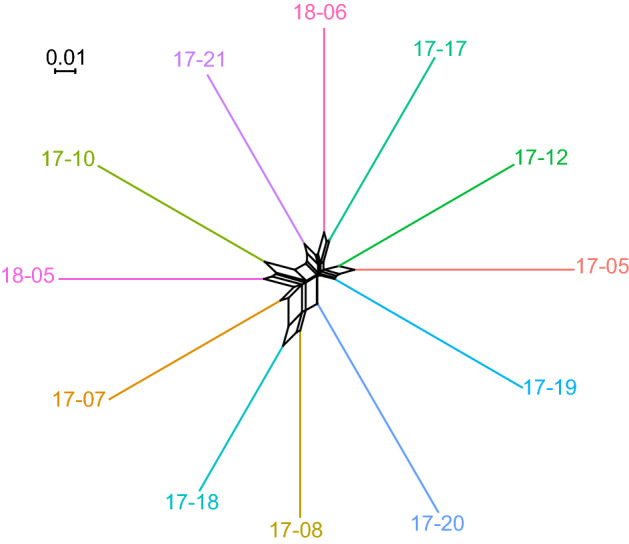
Phylogenetic network based on genomic diversity data for 12 bowhead whales from the EGSB stock indicating a star-like (branching-from-the-centre) pattern, but no structuring of more closely related individuals.

The PCA revealed no population structure within the EGSB stock (Fig. 2). This was supported by the phylogenetic network (Fig. 3), which showed a star-like (branching-from-the-centre) pattern, but no structuring of more closely related individuals.

### Relatedness and inbreeding

For the 12 bowhead whale individuals sampled in 2017 and 2018, coefficients of relatedness were all smaller than ~ 5% (Supplementary Table S2), suggesting that none of the individuals were closely related. Consistent with this result, none of the individuals sampled in 2006 or 2010 showed any signs of relatedness to the individuals collected in 2017 and 2018 that were analysed in the present study. However, six of the seven samples collected in 2006 (individuals A-F, Supplementary Table S1) showed high degrees of relatedness to each other, which is best explained by re-sampling of the same individual (see below). Coefficients of relatedness between these individuals ranged from 50.5% (as expected for parent–offspring or full siblings), to 87.6% (approaching monozygotic twins). Observed and expected homozygosity did not differ significantly in any individual (*p* < 0.066). The NGSrelate inbreeding coefficient for the 12 individuals was 0. ROHan did not find any significant ROH in any of the 12 individuals when using the default ROHan setting of 1 × 10^–5^ for the definition of a ROH. However, when increasing this to 5 × 10^–5^ there was some ROH in all individuals except for 17–18 and 17–19 (Supplementary Table S3).

### Genetic diversity

**Figure 4:**
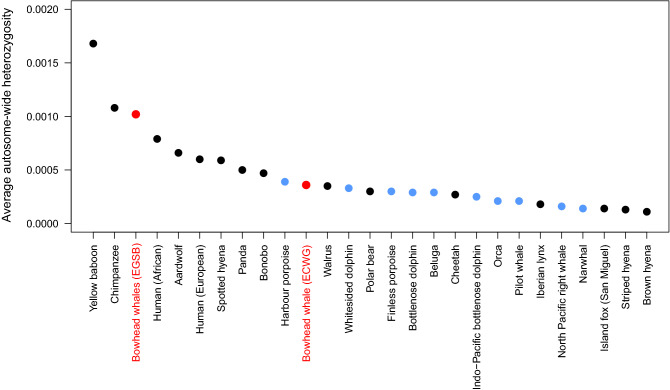
Mean autosome-wide heterozygosity proportions for EGSB stock bowhead whales (mean for seven individuals as listed in Table 1) compared to a variety of other mammalian species^36,36,42,43^ and the ECWG bowhead whale individual analysed by Keane et al*.*^22^, for which estimates are available calculated using the same software and parameters. Red dots show bowhead whales, blue dots other cetacean species and black dots other mammalian species.

The heterozygosity proportions (i.e. average proportion of sites within the autosomes that are heterozygous) ranged from 0.00098 to 0.00105 (Table 1) with a mean autosomal heterozygosity of 0.00102. A comparison of these heterozygosity estimates with those of a variety of mammalian species for which comparable data are available (Fig. 4) revealed that the EGSB stock bowhead whales display relatively high levels of heterozygosity. Furthermore, the EGSB individuals display heterozygosity proportions approximately three times higher than estimated for a conspecific from West Greenland^21^.

### Demographic analysis

**Figure 5:**
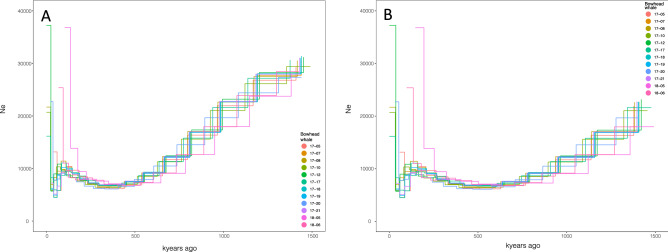
PSMC demographic analysis model outputs for 12 bowhead whales from the EGSB reaching back 1.5 million years. (**A**) using a generation time of 35 years. (**B**) using a generation time of 50 years.

Demographic analyses using PSMC suggested a more or less constant decrease in effective population size (*N*_e)_ from ca. 30,000 individuals some 1.5 million years ago to ca. 7000 individuals ca. 250,000 years ago. Subsequently, there was a slight increase of *N*_e_ up to ca. 9000 at 75,000 to 100,000 years ago. Figure 5 illustrates the demographic analysis for the last ca 1,500,000 years for both generation times of 35 and 50 years. Supplementary Table S4 provides the various estimates through time for a generation time of 35 years. For the period 1.5—5 million years ago, *N*_e_ was estimated as more or less stable between 20,000 and 30,000 individuals (Supplementary Fig. S1 and Table S4). The demographic histories of all 12 individuals included in this study demonstrated similar demographic trajectories, and supported the results of the population structure analyses that all samples arise from a single population.

**Figure 6:**
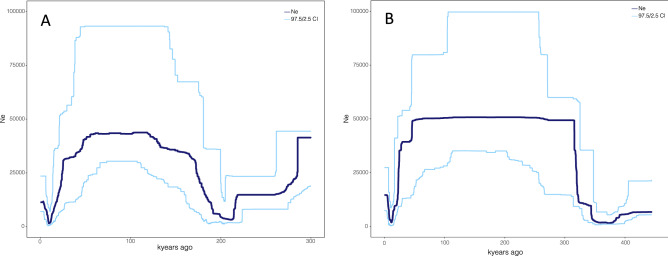
Stairway plot of the more recent demographic history of the EGSB stock with 2.5 and 97.5% confidence intervals. A: using an unfolded and B: using a folded site frequency spectrum as input. Generation time was set to 35 years.

The stairway plots (Fig. 6 and Supplementary Fig. S2) revealed several instances of change in *N*_e_. There was a decrease beginning approximately ~ 300,000 years ago that lasted approximately 100,000 years, followed by an increase over an approximately 100,000-year period beginning ~ 200,000 years (until 100,000 years ago), followed by a stable *N*_e_ until around 50,000 years ago before a dramatic decrease between 50,000 and 20,000 years ago. The plots were similar when using both the folded and unfolded SFS as inputs (Fig. 6 and Supplementary Fig. S2).

## Discussion

The potential consequences of more than three centuries of extensive whaling on the EGSB bowhead whale stock (previously known as the Spitzbergen stock) have been a concern raised by both researchers and conservation and management bodies such as the International Whaling Commission (IWC) and the IUCN^1–4,17,44^. In the Svalbard Archipelago, the hunt for bowhead whales started shortly after the discovery of the archipelago, and by the early nineteenth century few bowhead whales remained in the surrounding waters^45^. The EGSB stock became officially protected in 1932^45–47^. Recent studies suggest that the census size of the EGSB stock is at least in the low hundreds, and that it is either increasing, or has never been as low as the worst-case scenario of tens suggested based on decades with virtually no sightings^11,12^. Nevertheless, it is undisputed that population size is still far below the estimates calculated for the pre-whaling stock. Moreover, current climate change, and in particular sea ice declines, and Arctic marine traffic intensification may further negatively affect the already heavily depleted EGSB stock^1^.

Based on the analyses reported here, there are only a few significant runs of homozygosity (ROH) for the 12 individuals sampled in 2017 and 2018. Furthermore, autosomal heterozygosity proportion estimates for seven bowhead whales sampled in 2017/18 (Fig. 4) were relatively high compared to estimates for other mammalian species for which comparable estimates obtained by the same methods are available^36,38,42,43^. However, the bowhead whale is a long-lived mammal, and its longevity may extend past 200 years^21,48^. Even if hunting was extreme, the effective population size would have needed to be extremely small over the entire period of hunting to observe substantial reductions in genetic diversity in the present. The findings were somewhat different for six (samples A–F) of the seven individuals sampled in 2006, which showed very high relatedness values. However, as has been speculated earlier^19^, this 2006 collection is likely biased; all samples were collected within a very small area on the same day from a group of bowhead whales that were moving in synchrony^8^. Accordingly, re-sampling of the same individual may best explain the finding of identical mitochondrial genome sequences for the six individuals in question^18^. Importantly, the low coverage data for the samples collected in 2006 and 2010 were only used for the relatedness analysis; accordingly, the most likely duplicate samples did not bias any other analyses.

Low levels of relatedness between individuals and ROH, in addition to high levels of autosomal heterozygosity proportions, is often indicative of large population size. The most recent population estimate of ~ 350 individuals for EGSB bowheads, which is based on a combined helicopter- and ship-based line transect survey from the Russian border westward to the northwest corner of the Svalbard Archipelago stock, is still rather low^11^. It must be noted, however, that this estimate only represents part of the stock’s range, and the genomes of the sequenced individuals are highly diverse genetically speaking. Of note, autosomal heterozygosity levels were similarly high in all seven bowhead whale individuals analysed in this context. However, high genetic diversity does not always translate to large population size. Factors such as changes in population size, gene flow or inbreeding levels can also strongly affect the estimates. For various remnant populations of Cape buffalos (*Syncerus caffer*) and Giant panda (*Ailuropoda melanoleuca*), for example, relatively high genome-wide heterozygosity has been reported while population sizes are known to be low^49^. This may not be surprising because the population decline is recent in these cases. However, in other Arctic whale species such as the beluga whale (*Delphinapterus leucas*) and narwhal (*Monodon monoceros*), which both have a substantially larger population sizes, lower mean autosomal heterozygosity than the average EGSB bowhead whale value derived herein have been observed; for the narwhal, a recent rapid expansion from a much smaller founding population has been suggested as a likely driver of the low genetic diversity found for this species^38^. Unfortunately, except for the one ECWG bowhead whale individual sequenced by Keane et al*.*^22^, there are to date no other genome-wide estimates for individuals from other bowhead whale stocks. As pointed out above, it remains open as to what extent the estimates presented here reflect genetic diversity of bowhead whales across the entire distribution range of the species; though BCB whales have also retained high levels of diversity despite heavy historical harvests. Previous studies on mitochondrial genome diversity of contemporary EGSB stock individuals also indicated relatively high levels of genetic diversity (haplotype diversity (*H*_*D*_): 0.858, nucleotide diversity (*π*): 0.0027)^17,19^. Yet, continued very small population size, or even further decline in population size would mean an increasingly rapid accumulation of inbreeding and inbreeding depression.

When attempting to interpret the genetic diversity data in light of census and/or stock size of the EGSB stock, it has to be noted that census size estimates to date do not cover the entire range of this stock (see above). It is very unlikely that the stock consists of just one aggregation of animals moving as a group across the range. Rather, the stock probably segregates seasonally, and observers in different parts of the range see different feeding aggregations over the Arctic summer. Particularly little is known about bowhead whales in the western Russian Arctic, although bowheads are thought to reside in the Franz Josef Land region year-round^50,51^. Heide-Jørgensen et al*.*^52^ conclude that the waters around Franz Josef Land may be occupied by at least a hundred EGSB bowhead whales. Further east, bowhead whales have also been observed in the western Laptev Sea^53^, and the north of the Novosibirskie Islands Archipelago, which separates the Laptev and East Siberian seas^54^. Bowhead whales from the Bering-Chukchi-Beaufort (BCB) population are also believed to occasionally penetrate the East Siberian Sea, at least to 170° E longitude^55^. It is thus possible that individuals from the EGSB and BCB populations may meet in the Russian Arctic seas^52^. Briefly summarized, the knowledge on the current population size of the EGSB stock remains limited. The survey-based estimates of several hundred individuals are preliminary and further information is needed. The estimates of genetic diversity and an effective population size *N*_e_ in the low ten-thousands in the Pleistocene provide important information regarding EGSB stock size.

The demographic analyses of the genome data shed some light on the demographic trajectories through time of effective population size (*N*_e_) of the EGSB stock. They revealed a long-term (past ca. 1.5 M years), gradual decline in *N*_e_, a pattern similar to what has been reported for other baleen whale species^56^. Several fluctuations were also uncovered in the past 0.5 M years. In contrast, demographic analyses based on mitochondrial genomes from the same bowhead whale individuals sampled in 2017 and 2018 revealed a higher female effective population size (*N*_e(female)_) during the Pleistocene, ranging from ~ 70,000 to ~ 110,000 individuals^17^. However, as with genetic diversity estimates, estimates of *N*_e_ must be interpreted with caution; importantly, numerical estimates from different studies that are based on different genetic markers (nuclear vs mitochondrial genome) cannot be directly compared. Earlier studies have reported that the relative nuclear diversity of cetacean species is correlated with population size, while mitochondrial diversity is not^57–59^. For mitochondrial diversity, social structure and matrilineal social systems were emphasized as important drivers of range-wide and regional mitochondrial genetic diversity. Despite all these uncertainties, the mitochondrial genome analyses also supported an increase in *N*_e_ for EGBS bowhead whales during the late Pleistocene^17^, in agreement with the demographic pattern obtained in this study with nuclear genome data.

Estimates of *N*_e_ depend heavily on key parameters such as generation time and mutation rate as well as assumptions on gene flow. Accordingly, they cannot be considered exact values and must be interpreted with caution. The mutation rate of 7.69 × 10^–10^ substitutions per site per year for nuclear genomic DNA, which was used in the present study, and which was estimated specifically for bowheads^36^, may be considered low. It is substantially lower than the average mammalian genome mutation rate of 2.23 × 10^–9^ per site per year suggested by Kumar & Subramanian^60^. However, this discrepancy is not surprising, as the latter estimate was not based on genome-wide data, but rather on the analyses of some protein coding genes. The mutation rate used in the present study is in line with reduced substitution rates observed within Cetacea^61^, and is similar to the average nuclear mutation rate of 4.5 × 10^–10^ per site per year calculated by Jackson et al*.*^62^ for baleen whales. Although using another mutation rate would have resulted in different numerical estimates of *N*_e_, the order of magnitude of change in effective population size for the late Pleistocene EGSB stock estimated in the current study lies within the range of census size estimates based on information from other sources, for example, 25,000–100,000 individuals as suggested by Allen & Keay^3^. The extent of gene flow between stocks is a further uncertainty for estimates of *N*_e_. Given the long period of several millions of years encompassed by the demographic analysis, it is possible that free migration between stocks might have been possible in periods with minimal sea ice, as well as significant changes in ranges. Fossils of bowhead whales have, for example, been discovered along the Norwegian, Swedish and Danish coasts^63,64^, well south of the species' current distribution, indicating range shifts over time in response to climate change.

## Conclusion

Genetic differentiation and population sizes of historic and contemporary bowhead whale populations have been the subject of considerable research effort. Most molecular studies on these topics have used relatively short mitochondrial D-loop sequences to address levels of differentiation. Despite high rates of gene-flow between the EGSB, ECWG and BCB populations in the recent past^58^, small, but significant genetic differentiation between the four recognized stocks have been observed^14,16,17,65^. This study provides the first nuclear genomic baseline data set for the EGSB stock and reveals high genetic diversity in bowhead whales in this region. This information calls for more comprehensive analyses of genetic differentiation and for the reconstruction of the demographic history of bowhead whale stocks in the future.

## Supplementary Information

Supplementary Figure S1.

Supplementary Figure S2.

Supplementary Table S1.

Supplementary Table S2.

Supplementary Table S3.

Supplementary Table S4.

## Data Availability

Data are available as electronic supplementary material. The raw 150 bp paired-end Illumina NextSeq 500 reads are deposited in NCBI's Sequence Read Archives (Bioproject: PRJNA643010 Sand Bioproject: PRJNA798027).
