# Supplementary material for: High genomic diversity in the endangered East Greenland Svalbard Barents Sea stock of bowhead whales (Balaena mysticetus)
Source: Sci Rep. 2022 Apr 12;12:6118. doi: 10.1038/s41598-022-09868-5 (PMC9005726; doi:10.1038/s41598-022-09868-5)

**Supplementary Figure S1:** PSMC demographic analysis model outputs for 12 bowhead whales from the EGSB reaching back 5 million years. A: using a generation time of 35 years. B: using a generation time of 50 years.

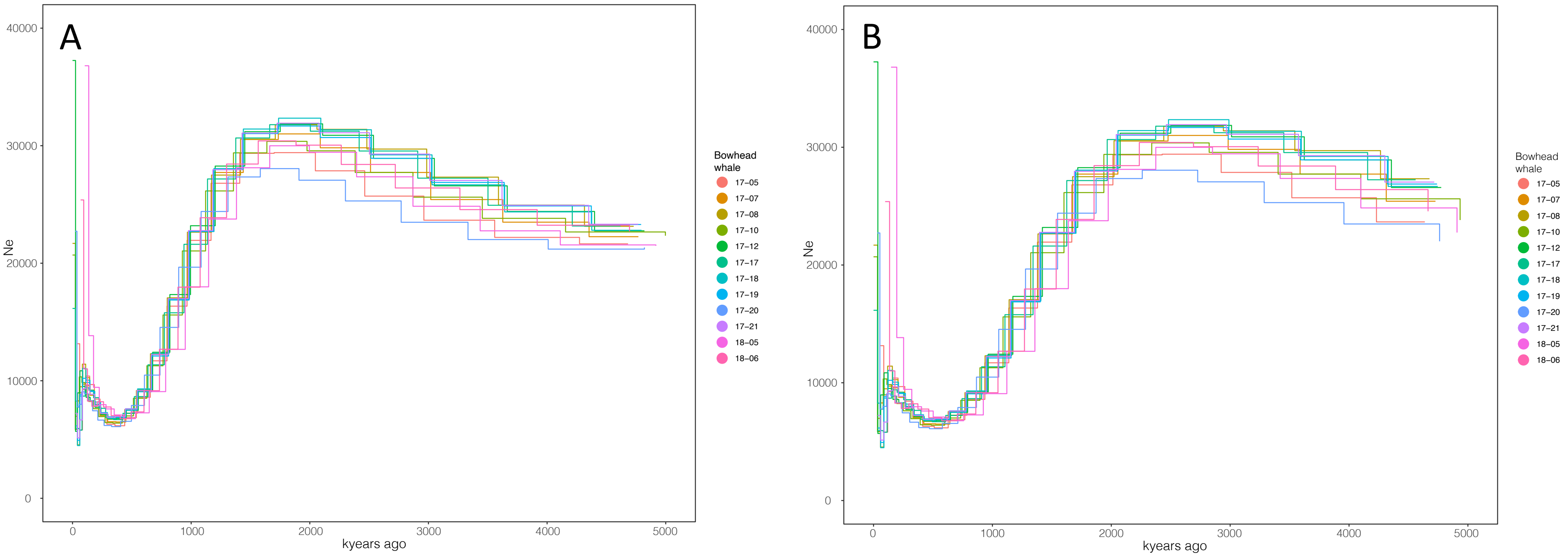

Supplement: Supplementary file 1 — Supplementary Figure S1. [file 41598_2022_9868_MOESM1_ESM.pdf]
