# Supplementary material for: High genomic diversity in the endangered East Greenland Svalbard Barents Sea stock of bowhead whales (Balaena mysticetus)
Source: Sci Rep. 2022 Apr 12;12:6118. doi: 10.1038/s41598-022-09868-5 (PMC9005726; doi:10.1038/s41598-022-09868-5)

Supplementary Figure S2: Stairway plot of the more recent demographic history of the EGSB stock with 2.5 and 97.5 % confidence intervals using a generation time of 50 years.

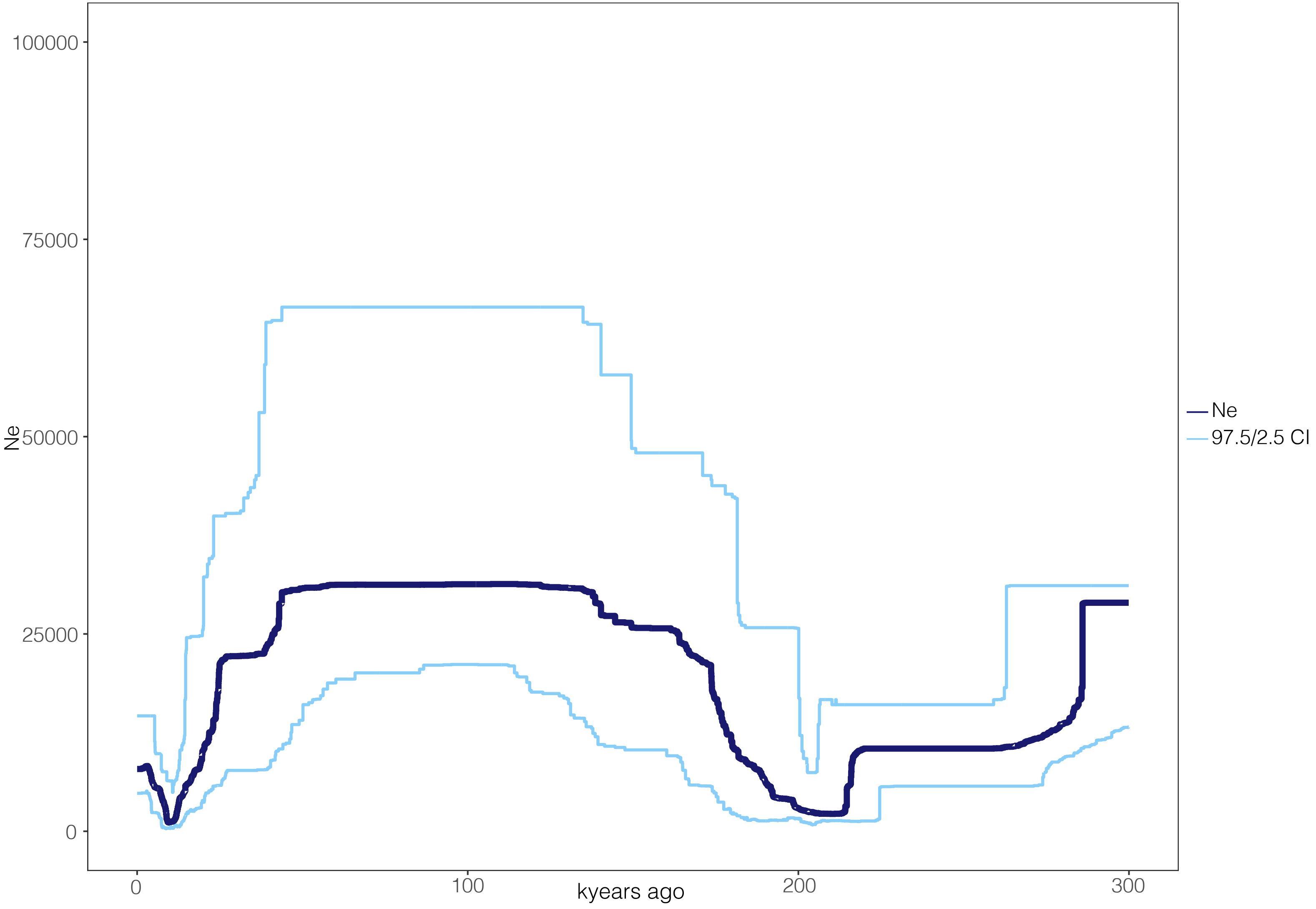

Supplement: Supplementary file 2 — Supplementary Figure S2. [file 41598_2022_9868_MOESM2_ESM.pdf]
