# Supplementary material for: High genomic diversity in the endangered East Greenland Svalbard Barents Sea stock of bowhead whales (Balaena mysticetus)
Source: Sci Rep. 2022 Apr 12;12:6118. doi: 10.1038/s41598-022-09868-5 (PMC9005726; doi:10.1038/s41598-022-09868-5)
Supplement: Supplementary file 3 — Supplementary Table S1. [file 41598_2022_9868_MOESM3_ESM.pdf]

Supplementary Table S1. EGSB bowhead whale stock samples included in the current study.

Sample ID	collected	sex	Latitude	Longitude	raw reads	Percent reads that mapped to reference	Coverage (ca. x)	SRA BioProjects: PRJNA643010 and PRJN798027 accessions
**17-05**	03.06.17	male	76.53N	3.54W	253,183,490	95.6	13.88	SRR15669496
**17-07**	04.06.17	female	76.41N	4.14W	365,705,208	95.9	20.22**	SRR15669495
**17-08**	04.06.17	male	76.41N	4.14W	439,217,342	94.9	23.74**	SRR15669492
**17-10**	04.06.17	female	76.34N	3.28W	300,910,478	95.8	16.54**	SRR15669491
**17-12**	04.06.17	female	76.35N	3.36W	425,564,908	95.7	23.22**	SRR15669490
**17-17**	05.06.17	male	76.34N	3.20W	403,224,222	95.2	21.94**	SRR15669489
**17-18**	05.06.17	male	76.29N	2.33W	406,088,430	95.7	20.88**	SRR15669488
**17-19**	08.06.17	male	76.50N	4.30W	499,791,902	95.2	20.68**	SRR15669487
**17-20**	08.06.17	female	76.50N	4.30W	264,272,696	95.5	14.02	SRR15669486
**17-21**	08.06.17	male	76.50N	4.31W	445,609,500	95.8	23.98**	SRR15669485
**18-05**	31.08.18	male	79.17N	18.25W	283,687,998	95.9	15.06	SRR15669494
**18-06**	31.08.18	male	79.17N	18.25W	302,575,026	95.8	16.06	SRR15669493
**A***	18.04.06	female	80.58N	2.09E	25,326,811		2.34	SRR17645797
**B***	18.04.06	female	80.58N	2.09E	22,803,823		2.27	SRR17645796
**C***	18.04.06	female	80.58N	2.09E	24,404,362		2.45	SRR17645795
**D***	18.04.06	female	80.58N	2.09E	24,528,479		1.72	SRR17645794
**E***	18.04.06	female	80.58N	2.09E	10,698,925		1.09	SRR17645793
**F***	18.04.06	female	80.58N	2.09E	26,861,564		0.8	SRR17645792
**I***	03.04.10	female	79.54N	1.03E	23,232,087		2.05	SRR17645791

* sequence data from Nyhus *et al.*[19] (for relatedness analysis only); ** samples used for calculation of autosome-wide heterozygosity
