# Supplementary material for: High genomic diversity in the endangered East Greenland Svalbard Barents Sea stock of bowhead whales (Balaena mysticetus)
Source: Sci Rep. 2022 Apr 12;12:6118. doi: 10.1038/s41598-022-09868-5 (PMC9005726; doi:10.1038/s41598-022-09868-5)
Supplement: Supplementary file 4 — Supplementary Table S2. [file 41598_2022_9868_MOESM4_ESM.pdf]

Supplementary Table S2: Relatedness levels between bowhead whale samples estimated using NGSrelate v2. Comparisons between individuals collected in 2017 and 2018 are shaded green, those including the individuals collected in 2006 and 2010 (Nyhus et al. 2016) are shaded grey.

**Bowhead whale**	**17-07**	**17-08**	**17-10**	**17-12**	**17-17**	**17-18**	**17-19**	**17-20**	**17-21**	**18-5**	**18-6**	**A**	**B**	**C**	**D**	**E**	**F**	**I**
**17-05**	0,000	0,000	0,000	0,012	0,010	0,000	0,024	0,000	0,018	0,000	0,000	0,000	0,000	0,000	0,000	0,000	0,000	0,000
**17-07**		0,000	0,000	0,000	0,000	0,019	0,006	0,000	0,000	0,015	0,000	0,000	0,000	0,000	0,000	0,000	0,000	0,000
**17-08**			0,005	0,000	0,000	0,053	0,000	0,001	0,000	0,000	0,000	0,000	0,000	0,000	0,000	0,000	0,000	0,000
**17-10**				0,011	0,000	0,000	0,000	0,000	0,013	0,036	0,000	0,000	0,000	0,000	0,000	0,000	0,000	0,000
**17-12**					0,000	0,000	0,000	0,000	0,000	0,000	0,000	0,000	0,000	0,000	0,000	0,000	0,000	0,000
**17-17**						0,000	0,000	0,000	0,006	0,000	0,050	0,000	0,000	0,000	0,000	0,000	0,000	0,000
**17-18**							0,000	0,000	0,000	0,000	0,000	0,000	0,000	0,000	0,000	0,000	0,000	0,000
**17-19**								0,000	0,000	0,011	0,000	0,000	0,000	0,000	0,000	0,000	0,000	0,000
**17-20**									0,000	0,000	0,000	0,000	0,000	0,000	0,000	0,000	0,000	0,000
**17-21**										0,000	0,030	0,000	0,000	0,000	0,000	0,000	0,000	0,000
**18-5**											0,000	0,000	0,000	0,000	0,000	0,000	0,000	0,000
**18-6**												0,000	0,000	0,000	0,000	0,000	0,000	0,000
**A**													0,860	0,865	0,859	0,835	0,540	0,000
**B**														0,876	0,869	0,841	0,505	0,000
**C**															0,874	0,847	0,513	0,000
**D**																0,840	0,518	0,000
**E**																	0,482	0,000
**F**																		0,000
