# Supplementary material for: High genomic diversity in the endangered East Greenland Svalbard Barents Sea stock of bowhead whales (Balaena mysticetus)
Source: Sci Rep. 2022 Apr 12;12:6118. doi: 10.1038/s41598-022-09868-5 (PMC9005726; doi:10.1038/s41598-022-09868-5)
Supplement: Supplementary file 5 — Supplementary Table S3. [file 41598_2022_9868_MOESM5_ESM.pdf]

Supplementary Table S3. Results of the ROHan analysis specifying a ROH if 1Mb of data contains an average proportion of heterozygous sites less than $5x10^{-5}$ for the definition of a ROH.

Sample	% of segments in ROH	Lower bound estimate	Upper bound estimate	Avg. length of ROH
**17-05**	0.1490	0.1490	0.1490	2,000,000
**17-07**	0.0746	0.0000	0.1493	1,000,000
**17-08**	0.5975	0.3734	0.9767	2,000,000
**17-10**	1.7280	0.9738	2.3538	1,916,670
**17-12**	0.1494	0.1493	0.2240	2,000,000
**17-17**	1.4211	1.0471	2.1164	1,900,000
**17-18**	0.0000	0.0000	0.0000	0
**17-19**	0.0000	0.0000	0.0000	0
**17-20**	0.5970	0.1491	0.8258	2,000,000
**17-21**	0.1490	0.1490	0.1490	2,000,000
**18-05**	0.6726	0.5966	1.1244	1,800,000
**18-06**	0.5220	0.2983	0.5232	1,750,000
