# Supplementary material for: High genomic diversity in the endangered East Greenland Svalbard Barents Sea stock of bowhead whales (Balaena mysticetus)
Source: Sci Rep. 2022 Apr 12;12:6118. doi: 10.1038/s41598-022-09868-5 (PMC9005726; doi:10.1038/s41598-022-09868-5)
Supplement: Supplementary file 6 — Supplementary Table S4. [file 41598_2022_9868_MOESM6_ESM.pdf]

Supplementary Table S4: The demographic histories (timeline and *Ne* estimates) of EGSB stock bowhead wha
The graphical illustration is presented in Figure 4 of the main manuscript.

timeline (years)

**ca.**	**30000**	**50000**	**75000**	**100000**	**150000**	**200000**	**250000**	**500000**	**750000**	**1000000**
**17_05**	28372	52741	73104	97535	143627	204198	254812	525840	778497	1038480
**17_07**	31155	48126	68456	106735	156919	198774	248910	516363	764665	1019479
**17_08**	30668	47408	78974	105340	155058	196586	246388	512822	760946	1016097
**17_10**	28460	52928	73386	97943	144303	205270	256247	529542	784645	1047369
**17_12**	31896	49274	70091	109293	160695	203569	254931	528975	783453	1044641
**17_17**	29376	45431	75736	101073	148900	211785	264360	494094	734129	1079009
**17_18**	31159	48165	80233	107015	157519	199699	250282	520864	772827	1031904
**17_20**	33170	48944	78745	103682	150796	212812	264706	491805	729497	1072384
**17_21**	30936	47821	79661	106254	156400	198284	248512	517512	767424	1024712
**18_05**	32233	49770	70760	95885	143148	205041	256613	480671	785668	1046444
**18_06**	31140	48122	80128	106844	157193	199221	249599	518793	769167	1026413
**17_19**	32785	50666	72099	97791	146208	209751	262792	493939	734708	982086
**average**	**30945**	**49116**	**75114**	**102949**	**151730**	**203749**	**254846**	**510935**	**763802**	**1035751**

estimated *Ne*

**17_05**	6955	4306	7271	8916	8256	7375	5791	7994	14824	24656
**17_07**	5013	7661	8771	8796	8082	7286	6611	8693	16214	21708
**17_08**	3293	9265	9754	9067	8585	7639	6853	8850	16336	21699
**17_10**	4402	10240	8826	7925	7526	6533	6096	8110	14745	24646
**17_12**	4670	9113	10139	8131	7869	7334	6828	8940	16773	22456
**17_17**	3323	6561	9096	8318	8357	7344	6666	8333	15072	26169
**17_18**	2564	9884	9823	8906	8555	7834	7045	8945	16185	21754
**17_20**	7256	7256	8111	7714	6571	6072	5783	7337	13212	22431
**17_21**	2778	8015	9358	8759	8168	7518	6990	8865	16236	21768
**18_05**	2332655	14879	12077	11558	6630	7215	7088	6780	15938	21158
**18_06**	12486	8984	6334	6803	8089	7861	7004	8658	16262	21582
**17_19**	6157	4929	8838	10208	9081	8008	7270	7505	12104	22709
**average**	**6157**	**8424**	**9033**	**8758**	**7980**	**7334**	**6668**	**8250**	**15325**	**22728**

ıles using a Pairwise Sequentially Markovian Coalescent (PSMC) model.

**1500000**	**2000000**	**3000000**	**4000000**	**5000000**
1516374	2008125	2912048	3842180	5064467
1486572	1965912	3118461	4105352	4928444
1484789	1966752	3128430	4125421	4958147
1530725	2028521	2944363	3887563	5127943
1523489	2014964	2916007	3840810	5053396
1577986	2090836	3034284	4005720	5283043
1507777	1997088	3176379	4188404	5033645
1567019	2076969	3016196	3984504	5259076
1497318	1983284	3154554	4159730	4999270
1523828	2013093	2908752	4191969	5028797
1498542	1983631	3151662	4153153	4989135
1577738	2088461	3025803	3988871	4792779
**1524346**	**2018136**	**3040578**	**4039473**	**5043178**

27953	26810	23086	20748	20870
29474	30033	24770	21521	20877
29585	30770	26905	22652	21561
29091	28609	24965	21829	21329
30354	30287	26131	23927	21148
31123	30754	26933	22668	21571
30628	30893	26253	22243	21504
26451	25792	21180	20281	21137
30225	30594	26575	22765	21788
28172	28598	23098	21444	20982
28595	27959	24691	23273	22246
31028	30694	26875	24874	23294
**29389**	**29316**	**25121**	**22352**	**21525**
